# The association between area deprivation and COVID-19 incidence: a municipality-level spatio-temporal study in Belgium, 2020–2021

**DOI:** 10.1186/s13690-022-00856-9

**Published:** 2022-04-02

**Authors:** Marjan Meurisse, Adrien Lajot, Brecht Devleesschauwer, Dieter Van Cauteren, Herman Van Oyen, Laura Van den Borre, Ruben Brondeel

**Affiliations:** 1 Department of Epidemiology and Public Health, Sciensano, Brussels, Belgium; 2 Department of Translational Physiology, Infectiology and Public Health, Ghent University, Merelbeke, Belgium; 3 Department of Public Health and Primary Care, Ghent University, Ghent, Belgium; 4 Interface Demography, Department of Sociology, Vrije Universiteit Brussel, Brussels, Belgium

**Keywords:** COVID-19, Incidence, Socio-economic inequality, Area deprivation, Belgium

## Abstract

**Background:**

In Belgium, current research on socio-economic inequalities in the coronavirus disease 2019 (COVID-19) crisis has mainly focused on excess mortality and data from the first epidemiological wave. The current study adds onto this by examining the association between COVID-19 incidence and area deprivation during the first five wave and interwave periods, thus adding a temporal gradient to the analyses.

**Methods:**

We use all confirmed COVID-19 cases between March 2020 and June 2021 in Belgium, aggregated at the municipality-level. These data were collected by the national laboratory-based COVID-19 surveillance system. A level of area deprivation was assigned to each Belgian municipality using data of three socio-economic variables: the share of unemployed persons in the active population, the share of households without a car and the share of low-educated persons. The spatio-temporal association between COVID-19 incidence and area deprivation was assessed by performing multivariate negative-binomial regression analyses and computing population attributable fractions.

**Results:**

A significant association between COVID-19 incidence and area deprivation was found over the entire study period, with the incidence in the most deprived areas predicted to be 24% higher than in the least deprived areas. This effect was dependent on the period during the COVID-19 crisis. The largest socio-economic inequalities in COVID-19 infections could be observed during wave 2 and wave 3, with a clear disadvantage for deprived areas.

**Conclusion:**

Our results provide new insights into spatio-temporal patterns of socio-economic inequalities in COVID-19 incidence in Belgium. They reveal the existence of inequalities and a shift of these patterns over time.

**Supplementary Information:**

The online version contains supplementary material available at 10.1186/s13690-022-00856-9.

## Background

The coronavirus disease 2019 (COVID-19) has led to a worldwide public health crisis since the outbreak in December 2019. Contrary to the initial belief that “we are all in this together”, international research reveals considerable social health disparities in COVID-19 infections and health outcomes [1–6]. There are indications of higher risks for exposure, infection, symptom severity, hospitalization and death among disadvantaged groups (e.g., low-income groups, people with a migrant background) and certain occupations (e.g., healthcare workers). Further research is needed to understand these patterns and how these develop throughout the different waves of the epidemic.

Belgium provides an interesting case from an international perspective as the country experienced a heavy burden of COVID-19. In April 2020, Belgium was one of three European Union member states that experienced a monthly excess mortality rate of more than 50%, i.e., 73% [7]. By the end of October 2020, the number of weekly new hospital admissions per 100,000 reached a value of 41, the third highest after the Czech Republic and Romania, and in November 2020 a second peak in monthly excess mortality of 59% was reached [7, 8]. Furthermore, Belgium already experienced large social disparities in both health status and health determinants prior to the COVID-19 outbreak [9, 10]. For example, people with a low education level were found to have a lower life expectancy and to suffer more frequently from chronic diseases compared to people with a high education level [9].

The COVID-19 crisis has been described as a syndemic, as it interacts with and is exacerbated by social, economic and health inequalities. Risk factors and comorbidities (e.g., obesity) are expected to be intertwined, interactive and cumulative [11, 12]. Current Belgian research on the association between socio-economic (SE) characteristics and COVID-19 has focused on excess mortality [13–16]. To date, there is only one other Belgian study on COVID-19 incidence during the first wave [17]. Therefore, we focus here on the socio-economic status (SES) as a potential societal determinant associated with COVID-19 incidence in Belgium over the period 1 March 2020 – 1 June 2021, covering three waves and two interwave periods.

In absence of individual data on SES, SE area deprivation is a well-established proxy for assessing the impact of SE characteristics on health [18–23]. These indices provide an area-level approximation of SE deprivation of individual inhabitants of that area and are composed of SE variables available for the total Belgian population. Furthermore, area-level deprivation indices capture social environmental factors operating in the environment of a person and outside a persons’ control, as described by Durkheim as the ‘social facts’ concept [24]. Using the municipality unit to assign a record to an area facilitates easy linking of COVID-19 health information to area deprivation levels. In addition, this approach allows for setting out community or regional directed policy measures. Inequalities in health across and within countries are the result of a complex interplay of SE determinants of health situated at different policy levels: international, national, regional, local, community-level, family-level and individual level. Our study focusses on SE inequalities on the municipality-level within Belgium, an interesting level for the development of focused health interventions [10, 25].

The aim of this manuscripts is to examine the spatio-temporal association between incidence of COVID-19 and area deprivation in Belgium. We investigate area deprivation as a possible explicative factor for higher COVID-19 incidence in a twofold way. First, we assess this association during different time periods of the COVID-19 crisis in Belgium, during which differences in the epidemiological situation, confinement measures and testing strategy were notable. Second, we look at both area deprivation as a whole and at the selected SE variables composing the index, to assess which variables were driving patterns of the association between area deprivation and area-level infection incidence. Building an understanding of where SE differences in COVID-19 come from and identifying societal risk factors are essential for making efficient public health decisions in the current and future pandemics.

## Methods

### Study design and area

This was an observational, retrospective study on Belgian COVID-19 confirmed cases, during the period 1 March 2020 to 1 June 2021, using aggregated data at the municipality level for the 581 Belgian municipalities.

### COVID-19 incidence

Data on COVID-19 incidence were collected through the national COVID-19 surveillance system, which was set up by Sciensano to monitor daily trends of virus circulation in the Belgian population [26]. Data on confirmed COVID-19 cases between 1 March 2020 and 1 June 2021 were included in the study. For each case record, information was collected such as the date on which the test was carried out, and the municipality of residence of the infected person. These case-based data were aggregated to obtain the number of confirmed COVID-19 cases by day and municipality.

**Fig. 1:**
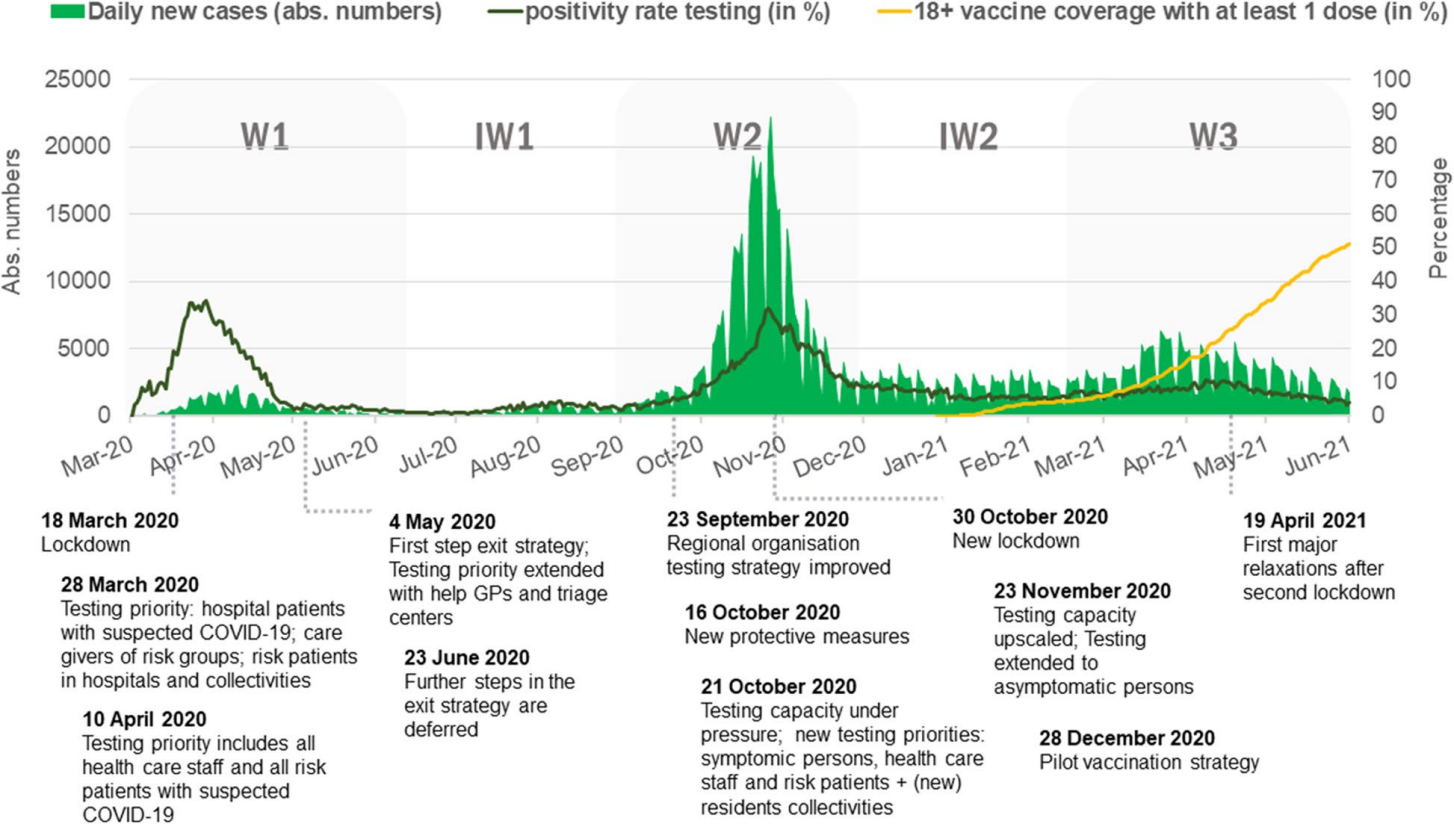
The evolution of the daily number of COVID-19 cases (left y-axis), test positivity rate (right y-axis) and vaccine coverage in the general 18-plus population with at least one dose in percentage (right y-axis), March 2020—June 2021, Belgium

Five periods of interest were distinguished based on differences in the epidemiological situation, confinement measures and testing strategy (Fig. 1): (1) wave one (W1, 1 March 2020 – 22 June 2020), (2) interwave one (IW1, 23 June 2020 – 30 August 2020), (3) wave two (W2, 31 August 2020 – 1 December 2020), (4) interwave two (IW2, 2 December 2020 – 14 February 2021) and (5) wave three (W3, 15 February 2021 – 1 June 2021). We calculated the COVID-19 incidence for each municipality over each of the time periods as the median number of cases by week, defined by the median of the number of cases each day of that period multiplied by 7. This resulted in one COVID-19 incidence value per municipality and per time period.

### Area deprivation index

The area deprivation index was built on the Carstairs deprivation index [19] and the Belgian adaptation of the index in quintiles by Hagedoorn et al. [18]. As in the method of Hagedoorn et al. [18], three variables approaching material deprivation were used for the calculation of the index: unemployment, not having a car, and low education level. Unemployment can affect health through its indirect link with a lack of material resources, social isolation and stress [27]. Car ownership was used as an indication of wealth. It could also be linked to the urban context in which people live and the frequency with which people use public transport. It therefore potentially has a link with the risk of infection. Low education was used as the last variable, indicating low socio-economic status. It is associated with employment and income, links to health literacy and problem coping [28], and has the ability to capture constraints of SE conditions during childhood [27]. Low social class and overcrowding as used in the Carstairs deprivation index were not included, as data were not available.

Data from the ‘Vlaamse Arbeidsrekening’ of the year 2018 were used to derive unemployment information for all Belgian municipalities [29]. This was measured as the percentage of the active population between 15 and 64 years old that was unemployed. The Belgian statistical office, STATBEL, provided information on car ownership for the year 2019 [30], calculated as the percentage of households in a municipality without a car. In addition, STATBEL data provided information on the educational level in 2017. The variable ‘low education level’ was calculated as the percentage of the population with a low education level as the highest educational achievement. A low education level was defined by the International Standard Classification of Education (ISCED) levels 0–2, which indicates a highest educational achievement less than primary, primary (6 years) or lower secondary education (entry after 6 years of primary education and end after 9 years since the beginning of primary education).

A level of area deprivation was assigned to each Belgian municipality. The three variables were combined into one score by summing up the z-scores of the component variables by municipality. Based on this combined score, municipalities were classified into quintiles (Q1-Q5), with Q1 the least deprived and Q5 the most deprived municipalities. Additionally, each municipality was classified into quintiles (Q1-Q5) based on the z-scores of the individual component variables (unemployment, not having a car, and low education level), resulting in a separate index for each SE component of the area deprivation index. This approach allows us to gain insights into the spectrum of area deprivation with five groups ranked from least to most deprived municipalities, as well as better understand potential differences for each SE component.

### Statistical analyses

#### Regression analysis

The association between COVID-19 incidence and area deprivation was assessed by using generalized linear models with a logarithmic link function, since our response variable COVID-19 incidence was a count outcome. First, a model was built with the area deprivation index as an explanatory variable and COVID-19 incidence as the outcome variable. Second, the period was additionally considered in interaction with the area deprivation index. In this way, it was explored whether the relationship between the area deprivation index and COVID-19 incidence depends on the time period of the COVID-19 crisis. Third, separate models were constructed for every individual SE indicator composing the area deprivation index: unemployment, not having a car, and low education level. All the of the above models were fitted using a negative binomial regression model, after overdispersion was detected with dispersion tests for equidispersion in the initially fitted Poisson regression models.

The log of the municipality-specific population at 1 January 2020 was used as an offset variable [31]. We controlled for the median age per municipality, and the 2019 degree of urbanization as operationalized by Eurostat [32] in every of the above-mentioned models.

Marginal effects were calculated to better understand the relationship between the area deprivation index and COVID-19 incidence. As a result, we could estimate the direct effect of a change in area deprivation on incidence.

All analyses were performed in R version 4.0.5 [33]. Negative-binomial models were fitted, overdispersion tests were executed and marginal effects were calculated using the R packages ‘MASS’, ‘AER’ and ‘effects’ respectively [34].

#### Population attributable fraction

The population attributable fraction (PAF) assesses the health impact of exposure to certain risks in a population [35, 36]. In this context, the PAF indicates the estimated proportion of the total COVID-19 incidence that would have been avoided if all municipalities would be classified in the least deprived quantile (Q1). The PAF was calculated using results from the regression model.

For each period, the PAF was calculated as the ratio between, on the one hand, the sum of the excesses of the expected incidence (EI) of each quintile (Q2, Q3, Q4 and Q5) compared to Q1 and, on the other hand, the sum of the expected incidence of all quintiles (Eq. 1).

1 $$100*\frac{(EI\left(Q2\right)-EI\left(Q1\right))+(EI\left(Q3\right)-EI\left(Q1\right))+(EI\left(Q4\right)-EI\left(Q1\right))+(EI\left(Q5\right)-EI\left(Q1\right))}{EI\left(Q1\right)+EI\left(Q2\right)+EI\left(Q3\right)+EI\left(Q4\right)+EI(Q5)}$$

Calculating confidence intervals was possible through a Monte Carlo simulation as we know the parameters of the normal distribution of the expected incidence of each quintile. For each period, 1000 iterations of the expected incidence were generated and for each of them the PAF was calculated. The 2.5 and 97.5 percentiles (median of the iterations) resulted in our confidence bounds.

## Results

Between 1 March 2020 and 1 June 2021, 1,062,680 confirmed COVID-19 cases were recorded in Belgium. Considerable differences were apparent in COVID-19 incidence during this period. Overall, Belgium had a median COVID-19 incidence of 21 for all municipalities. When considering (inter)wave periods, we found that the median COVID-19 incidence was 7 (W1 and IW1), 28 (W2), 14 (IW2) and 21 (W3). Across municipalities, the median percentages of unemployment, households without a car and population with a low education level equaled 6.8%, 17.2% and 37.2%, respectively.

The visualization of the COVID-19 incidence per 100,000 inhabitants, by the area deprivation (Supplementary Fig. 1) roughly shows a relationship between both variables and a dependence of this relationship on the degree of urbanization. The proportion of municipalities with each degree of urbanization was not homogeneous from one quintile to another, especially for Q5 – comprising the most deprived municipalities – that was presenting a high proportion of densely populated municipalities, while the proportion of rural areas was lower than for other quintiles (Supplementary Fig. 2). Furthermore, we observed that the age composition was not homogeneous from one quintile to another. Municipalities with a higher area deprivation tended to have a younger age structure.

**Fig. 2:**
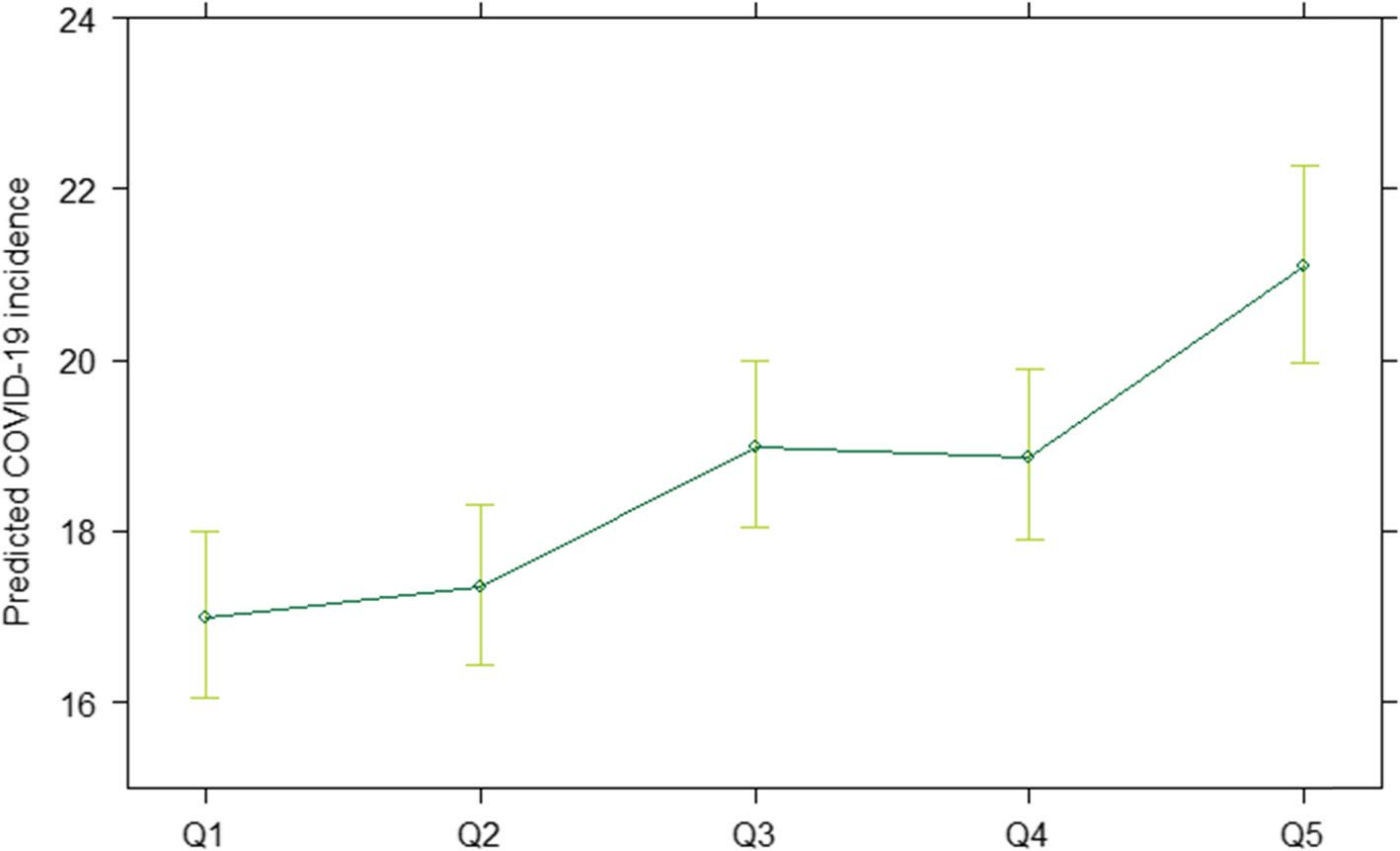
Predicted COVID-19 incidence (1 March 2020 – 1 June 2021, Belgium) from the negative binomial regression in function of the area deprivation index quintile (Q1-Q5). Q1 represents the least deprived quintile, Q5 the most deprived quintile. Municipality-specific population size was used as an offset variable and we controlled for the median age per municipality and degree of urbanization in the regression model

The first regression model, controlling for the median age per municipality and degree of urbanization but without considering the time period of the COVID-19 crisis, showed a significant association between COVID-19 incidence and the area deprivation index. The marginal effect of the area deprivation index on COVID-19 incidence over the whole observation period is depicted in Fig. 2. The predicted COVID-19 incidence was positively associated with the area deprivation index. The incidence in the most deprived municipalities (Q5) was predicted to be 24.1% (95% CI [14.6, 34.5]) higher than in the least deprived municipalities (Q1).

**Fig. 3:**
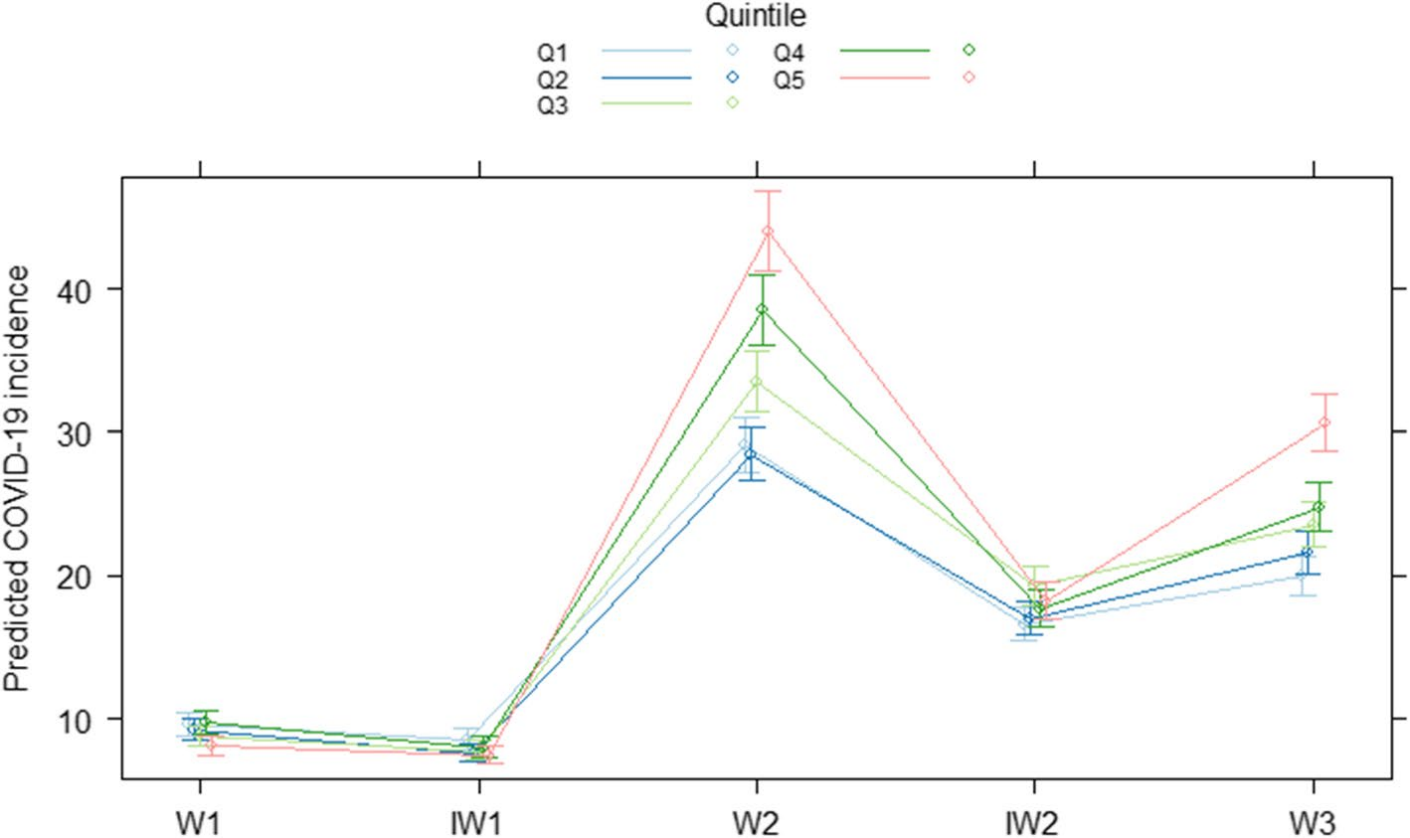
Predicted COVID-19 incidence from the negative binomial regression with the area deprivation index (Q1-Q5) as explanatory variable, by area deprivation, by period of interest in the Belgian COVID-19 crisis. W1: wave 1, 1 March 2020 – 22 June 2020; IW1: interwave 1, 23 June 2020 – 30 August 2020; W2: wave 2, 31 August 2020 – 1 December 2020; IW2: interwave 2, 2 December 2020 – 14 February 2021; W3: wave 3, 15 February 2021 – 1 June 2021. Municipality-specific population size was used as an offset variable and we controlled for the median age per municipality and degree of urbanization in the regression model

The second regression model, considering the time period of the COVID-19 crisis, indicated that the interaction between the period and the deprivation index was significant. In other words, the effect of area deprivation on COVID-19 incidence differed significantly across periods. As shown in Fig. 3, significant differences between the quintiles could be observed during W2, except between Q1 and Q2. The predicted COVID-19 incidence converged during IW2, after which differences between quintiles increased again in W3, although not to the same extent as observed in W2. Globally, for those periods, the most deprived municipalities were predicted to have a higher COVID-19 incidence than the least deprived municipalities. The model predicted that incidence in Q5 was 51.3% (95% CI [38.3, 65.9]), 9.8% (95% CI [-0.7, 21.6]) and 53.9% (95% CI [39.9, 69.5]) higher compared to Q1 during respectively W2, IW2 and W3. The association between COVID-19 incidence and the degree of urbanization remained significant, even when controlling for the median age per municipality and area deprivation, with a lower predicted incidence rate of COVID-19 cases in more densely populated areas (Supplementary Fig. 3).

**Fig. 4:**
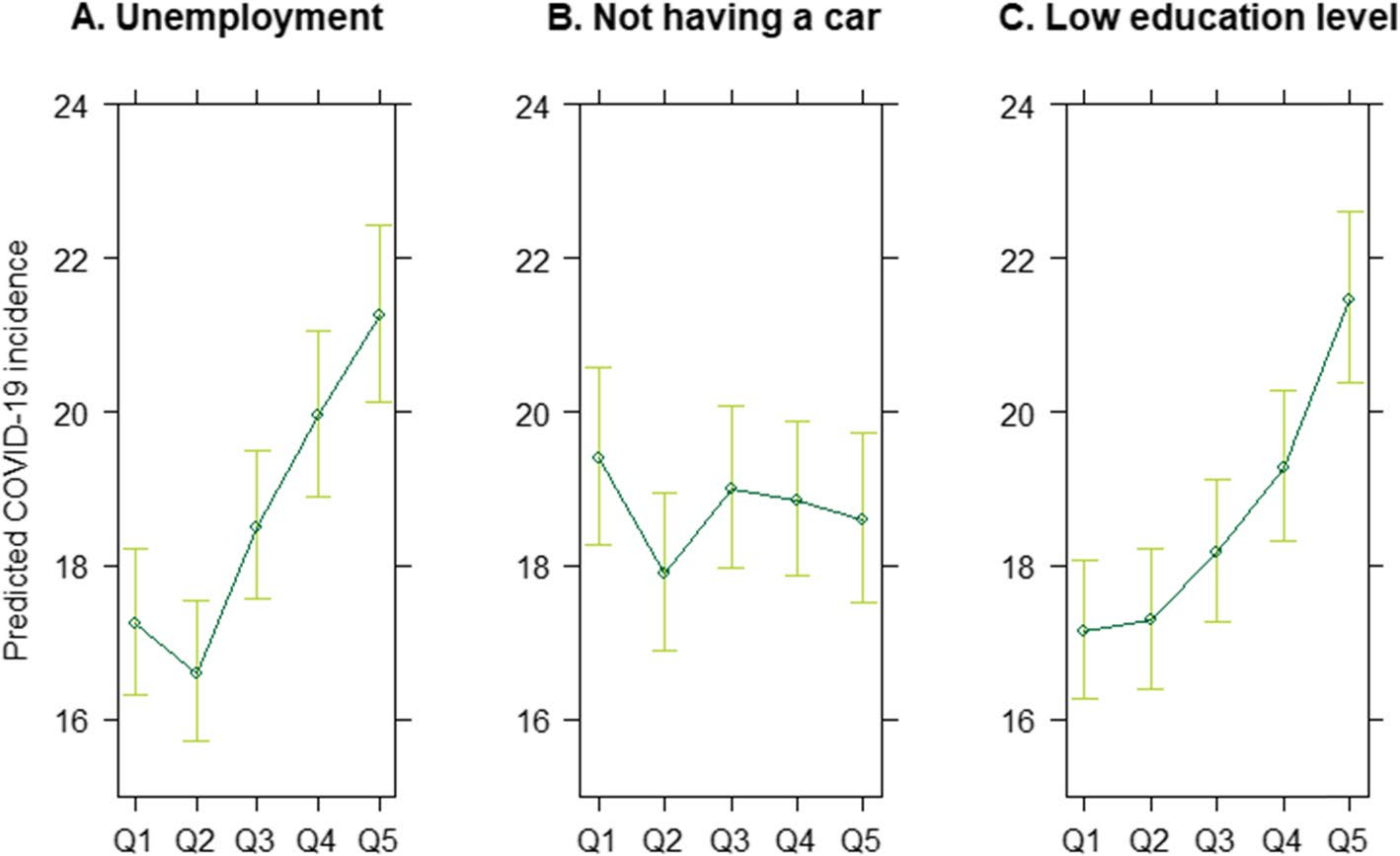
Predicted COVID-19 incidence (1 March 2020—1 June 2021, Belgium) from the negative binomial regression in function of the indices of SE indicators composing the area deprivation index (unemployment, not having a car, and low education level). The indices range from Q1 to Q5, with Q1 representing the least deprived quintile, and Q5 the most deprived quintile. Municipality-specific population size was used as an offset variable and we for the median age per municipality and degree of urbanization in the regression model

When investigating each individual SE indicator of the area deprivation index (unemployment, not having a car, and low education level) a significant association was found between COVID-19 incidence and two indices: unemployment and low education. Panes A and C in Fig. 4 indicate a higher predicted COVID-19 incidence in municipalities with higher levels of unemployment and in municipalities with a larger percentage of people with a low education level. However, no significant association was found between COVID-19 incidence and the index constructed of percentage households without a car (Fig. 4B).

For the calculation of the PAFs we contrasted the predicted incidence of the least deprived areas in Q1 with all the other quintiles (Q2-Q5), representing exposure in more deprived areas. In order to gain insight into the share of COVID-19 incidence that could be attributed to exposure in more deprived areas, Table 1 shows the PAF-values per period and the corresponding confidence intervals.

**Table 1:** Population Attributable Factor (PAF) calculated from the negative binomial regression by period of interest in the Belgian COVID-19 crisis. Entire period: 1 March 2020 2021 – 1 June 2021, Wave 1: 1 March 2020 – 22 June 2020; Interwave 1: 23 June 2020 – 30 August 2020; Wave 2: 31 August 2020 – 1 December 2020; Interwave 2: 2 December 2020 – 14 February 2021; Wave 3: 15 February 2021 – 1 June 2021. PAF = Population Attributable Fraction

Period	PAF	95% CI
Entire period	8.91	[8.79, 9.05]
Wave 1	-6.17%	[-6.50, -5.86]
Interwave 1	-8.24%	[-8.60, -7.89]
Wave 2	16.18%	[16.02, 16.37]
Interwave 2	6.59%	[6.35, 6.83]
Wave 3	17.34%	[17.14, 17.53]

During W1 and IW1 the PAF was negative, which means that exposure to a higher levels of area deprivation would decrease incidence for those periods, however, only by 6.2% and 8.2%, respectively. For W2, IW2 and W3, respectively, results show that 16.2%, 6.6% and 17.3% of the COVID-19 incidence is attributable to the exposure to a higher area deprivation.

## Discussion

This study examined the spatio-temporal association between COVID-19 incidence and area deprivation in Belgium by performing regression analyses and calculating PAFs. Our study adds onto research on SE inequalities in the COVID-19 crisis in Belgium by focusing on confirmed COVID-19 cases, covering a large period of time and adding a temporal gradient to the analyses.

Municipality-level SE inequalities were found in COVID-19 incidence within Belgium. Overall, more deprived municipalities were predicted to experience a higher incidence. This finding is consistent with earlier findings on infectious diseases [37, 38], in general, and on COVID-19, specifically. Prior international research on spatio-temporal differences in COVID-19 incidence has established a positive relationship with area deprivation in Louisiana (USA) [39], Utah (USA) [40], Kolkata city (India) [41], Chennai city (India) [42] and Italy [43]. High COVID-19 incidence has also been related to specific ecological indicators for the socio-economic profile of areas, such as educational attainment [1] and income [44].

There are several potential explanations for the observed association between COVID-19 incidence and area deprivation. When looking at the separate components of the area deprivation index, we found that the observed pattern was mainly driven by differences in the degree of unemployment and the percentage of the population with a low education level. Unemployment might cause a disproportionate burden of COVID-19 cases through possible negative consequences of unemployment, e.g., increased vulnerability due to pre-existing health problems or engagement in unhealthy behaviors [45]. A lower education level might lead to a higher incidence through e.g., concentration in low occupation jobs, in which people are more exposed to precarious working conditions associated with an increased risk of infection [12, 46, 47], or through differences in health literacy [48–50]. The index based on car ownership was not found to be associated with COVID-19 incidence. It is possible that, contrary to our hypotheses, car ownership is not linked to a higher frequency of public transport use and increased risk of infection in periods of lockdown and low general mobility. Previous research also suggests potential differences related to the urban–rural context in which people are living, potential economic stress related to car ownership and variations in the perceived value of having a car [51–53].

We observed however that this pattern of municipality-level SE inequalities was dependent on the period during the COVID-19 crisis. In W1 and IW1, no clear differences in predicted incidence between the quintiles could be observed. When considering the PAFs in these periods, small “gains” from living in a disadvantaged area could even be observed. However, in this period, testing was focused exclusively on severe cases, which doesn’t correctly reflect the real incidence [54]. In contrast, we found a significant difference in predicted incidence between most quintiles in W2 and W3 – periods associated with an intense circulation of the virus – with most deprived quintiles showing the highest predicted incidence. Less pronounced differences between quintiles were observed in IW2. Those findings were confirmed by the PAF-analysis, with an important percentage of the COVID-19 incidence attributable to higher deprivation in W2 and W3, and to a lesser extent in IW2. Similar results were found in Italy, where they only observed an association between COVID-19 incidence and area deprivation in the lockdown and post-lockdown periods [43]. In addition, Clouston and colleagues find similar changes in SE differences in COVID-19 incidence over time for the United States [5]. In line with the fundamental cause theory (FCT) [55–57], they hypothesize that the increased SE differences in COVID-19 incidence in the beginning of the epidemic may be the result of unequal diffusion of knowledge of risk factors and the unequal resources to implement the knowledge into health-protective behaviors. Insights into COVID-19 risk factors and protective measures may have been unevenly diffused through the social space, first reaching the most privileged. Throughout the course of the epidemic, SE differences in COVID-19 incidence were observed to converge, possibly as a result of increased knowledge transfer and policy measures affecting all layers of the population.

Another potential explanation for the change in SE differences over time may lie in the unique combination of testing criteria, social distancing measures and levels of viral circulation during the different considered periods. In W1 and part of IW1, testing criteria were limited. Only from 8 May 2020 onwards all symptomatic cases that fit with the case definition could be tested. Testing of high-risk contacts was only possible from 13 July 2020 onwards [54]. In W2 (except between 21 October 2020 and 23 November 2020 [54]), IW2 and W3, the testing strategy included all symptomatic cases that fit the case definition and high-risk contacts of COVID-19 cases. Moreover, in the interwave periods, viral circulation was relatively low compared to the epidemiological wave periods. Further, different lockdown periods were instated over the course of the study period. The association between COVID-19 incidence and area deprivation observed here, is most likely the result of a complex interplay of various interdependent elements, that shift over time.

Besides the effect of area deprivation, a significant effect of degree of urbanization on COVID-19 incidence was additionally found. More densely populated areas were predicted to experience a lower incidence. In theory, we would expect the opposite, i.e., a lower incidence in rural areas, as a lower population density would potentially reduce contact rates and thereby reduce the risk of COVID-19 transmission [58] and as high population density has been identified as a risk factor in previous studies [59–61]. However, in rural areas there might be a false perception of security and less control on enforced social-distancing measures, resulting in more risky behavior. Huang et al. [62] previously found a similar pattern, and observed lower case rates in urban areas compared to rural areas. However, they relate this pattern to reduced testing and contact tracing in rural areas, which in Belgium seems less plausible as sampling for tests can be performed at the GP and at geographically spread sampling centers [63]. True disentanglement of this pattern needs further investigation.

The use of the area deprivation index and aggregated data is presented with some limitations. First of all, when using the index, it is assumed that areas are internally homogeneous, which might not always be the case, e.g., municipalities with a combination of households with high and low deprivation will obtain a middle ranking score. Using the area deprivation index also makes this study susceptible to the ecological fallacy. The ecological fallacy results from the assumption that inferences can be made about individual patterns based on patterns observed in groups, which might not always be a correct assumption [64, 65]. Patterns observed on the municipal level might be different from patterns at the individual level [13]. Therefore, we remain careful in drawing conclusions at the individual level and focus on deprivation in areas instead of people. It is also important to emphasize that there is no ‘true’ definition of deprivation, there are many ways to measure its value. Our area deprivation index is an estimation of an area’s material deprivation and doesn’t take into account social deprivation, as material deprivation is speculated to be an easier target for policy measures [27]. The use of an area deprivation index also has multiple advantages, like the lack of a need for individual data and its relatively simple calculation. Working on an aggregated municipality-level also allows the identification of areas at risk for an increased COVID-19 incidence, which can be targeted for specific local policy measures.

Further, the use of PAFs is limited by not allowing comparison across different levels of area deprivation. The artificial nature of the PAFs has been criticized previously [66, 67]. However, the use of PAFs provides us with an uncomplicated inequality measure. It is translatable to policy measures by addressing the population impact of inequalities in area deprivation and the potential gain by instating policy measures that aim to reduce these inequalities [15]. Our results demonstrate that the PAFs can present a useful instrument to gain insights into the relative burden of living in a deprived area.

This manuscript provides a stepping-stone to lower-level analyses in future research, like statistical sector, i.e., the smallest administrative level, and individual level. Analyses at the statistical sector would reduce heterogeneity within the unit of area, while preserving the possibility to identify areas at risk to target for policy measures. Further, an examination of the drivers of the observed socio-economic inequalities, would be an important contribution in future research. Hence, examining cause-specific inequalities might be a valuable addition to the current study [68].

Our results provide valuable insights into spatio-temporal patterns of SE inequalities in COVID-19 incidence in Belgium. The large majority of studies on COVID-19 and SE differences in Belgium are based on excess mortality and these studies indicate the existence of SE inequalities during the first Belgian COVID-19 epidemiological wave [13, 15–17]. Our study is the first in Belgium that focusses on SE inequalities in COVID-19 cases over a larger period of time, also taking into account a temporal gradient. Our study shows the existence of inequalities in COVID-19 incidence and a shift of these patterns with time. It therefore corroborates the earlier scientific evidence in showing that “we are not all in this together”. Examination of underlying trends and risk factors should be explored further and can provide useful information for policy-making.

## Supplementary Information

**Additional file1: Supplementary figure 1.** Boxplots of the COVID-19 incidence rate (1 March 2020 – 1 June 2021, Belgium), or median weekly number of COVID-19 cases per 100,000 inhabitants, with the area deprivation by (A) median age interval for each quintile (Q1-Q5), (B) the percentage of people older than 60 years old within a given interval for each quintile (Q1-Q5) and (C) the degree of urbanization for each quintile (Q1-Q5) . Q1 represents the least deprived quintile, Q5 the most deprived quintile. *An outlier was removed of a municipality of Q1, with a COVID-19 incidence rate value of 8860,8. **Supplementary figure 2.** The proportion of Belgian municipalities (A) within a given median age interval for each quintile (Q1-Q5), (B) for which the percentage of people older than 60 years old is within a given interval for each quintile (Q1-Q5) and (C) with a given degree of urbanization for each quintile (Q1-Q5). Q1 represents the least deprived quintile, Q5 the most deprived quintile. **Supplementary figure 3.** Predicted COVID-19 incidence (1 March 2020 - 1 June 2021, Belgium) from the negative binomial regression in function of the degree of urbanization (i.e., Densely populated areas, Intermediate density areas and Rural areas). Municipality-specific population size was used as an offset variable and we controlled for the median age per municipality and area deprivation in the regression model.

## Data Availability

The datasets supporting the conclusions of this article are available in the Epistat repository. (https://epistat.wiv-isp.be/covid/), on the website of ‘Steunpunt Werk’ (https://www.steunpuntwerk.be/node/2779) and on the website of STATBEL (https://statbel.fgov.be/en/open-data/number-cars-statistical-sector). Data on education level at the municipal level were obtained through a direct data request from STATBEL.

## Abbreviations

COVID-19: Coronavirus disease 2019
SE: Socio-economic
SES: Socio-economic status
SARS-CoV-2: Severe acute respiratory syndrome coronavirus 2
W1: Wave one
IW1: Interwave one
W2: Wave two
IW2: Interwave two
W3: Wave three
PAF: Population attributable fraction
EI: Expected incidence
CI: Confidence interval
FCT: The fundamental cause theory
