# Correction to: The association between area deprivation and COVID-19 incidence: a municipality-level spatio-temporal study in Belgium, 2020–2021

**DOI:** 10.1186/s13690-022-00883-6

**Published:** 2022-04-29

**Authors:** Marjan Meurisse, Adrien Lajot, Brecht Devleesschauwer, Dieter Van Cauteren, Herman Van Oyen, Laura Van den Borre, Ruben Brondeel

**Affiliations:** 1 Department of Epidemiology and Public Health, Sciensano, Brussels, Belgium; 2 Department of Translational Physiology, Infectiology and Public Health, Ghent University, Merelbeke, Belgium; 3 Department of Public Health and Primary Care, Ghent University, Ghent, Belgium; 4 Interface Demography, Department of Sociology, Vrije Universiteit Brussel, Brussels, Belgium

**Correction to: Archives of Public Health 80, 109 (2022)**

**https://doi.org/10.1186/s13690-022-00856-9**

Following publication of the original article [1], due to a typesetting error, the sentence below was missing in the article.

Marjan Meurisse and Adrien Lajot contributed equally to this work.

The correct information has been provided in this Correction and the original article [1] has been updated.

## References

[1] Meurisse M, Lajot A, Devleesschauwer B (2022). The association between area deprivation and COVID-19 incidence: a municipality-level spatio-temporal study in Belgium, 2020–2021. Arch Public Health.

