# Supplementary material for: The association between area deprivation and COVID-19 incidence: a municipality-level spatio-temporal study in Belgium, 2020–2021
Source: Arch Public Health. 2022 Apr 2;80:109. doi: 10.1186/s13690-022-00856-9 (PMC8976211; doi:10.1186/s13690-022-00856-9)
Supplement: Supplementary file 1 — Additional file1: Supplementary figure 1. Boxplots of the COVID-19 incidence rate (1 March 2020 – 1 June 2021, Belgium), or median weekly number of COVID-19 cases per 100,000 inhabitants, with the area deprivation by (A) median age interval for each quintile (Q1-Q5), (B) the percentage of people older than 60 years old within a given interval for each quintile (Q1-Q5) and (C) the degree of urbanization for each quintile (Q1-Q5) . Q1 represents the least deprived quintile, Q5 the most deprived quintile. *An outlier was removed of a municipality of Q1, with a COVID-19 incidence rate value of 8860,8. Supplementary figure 2. The proportion of Belgian municipalities (A) within a given median age interval for each quintile (Q1-Q5), (B) for which the percentage of people older than 60 years old is within a given interval for each quintile (Q1-Q5) and (C) with a given degree of urbanization for each quintile (Q1-Q5). Q1 represents the least deprived quintile, Q5 the most deprived quintile. Supplementary figure 3. Predicted COVID-19 incidence (1 March 2020 - 1 June 2021, Belgium) from the negative binomial regression in function of the degree of urbanization (i.e., Densely populated areas, Intermediate density areas and Rural areas). Municipality-specific population size was used as an offset variable and we controlled for the median age per municipality and area deprivation in the regression model. [file 13690_2022_856_MOESM1_ESM.docx]

# Supplementary information


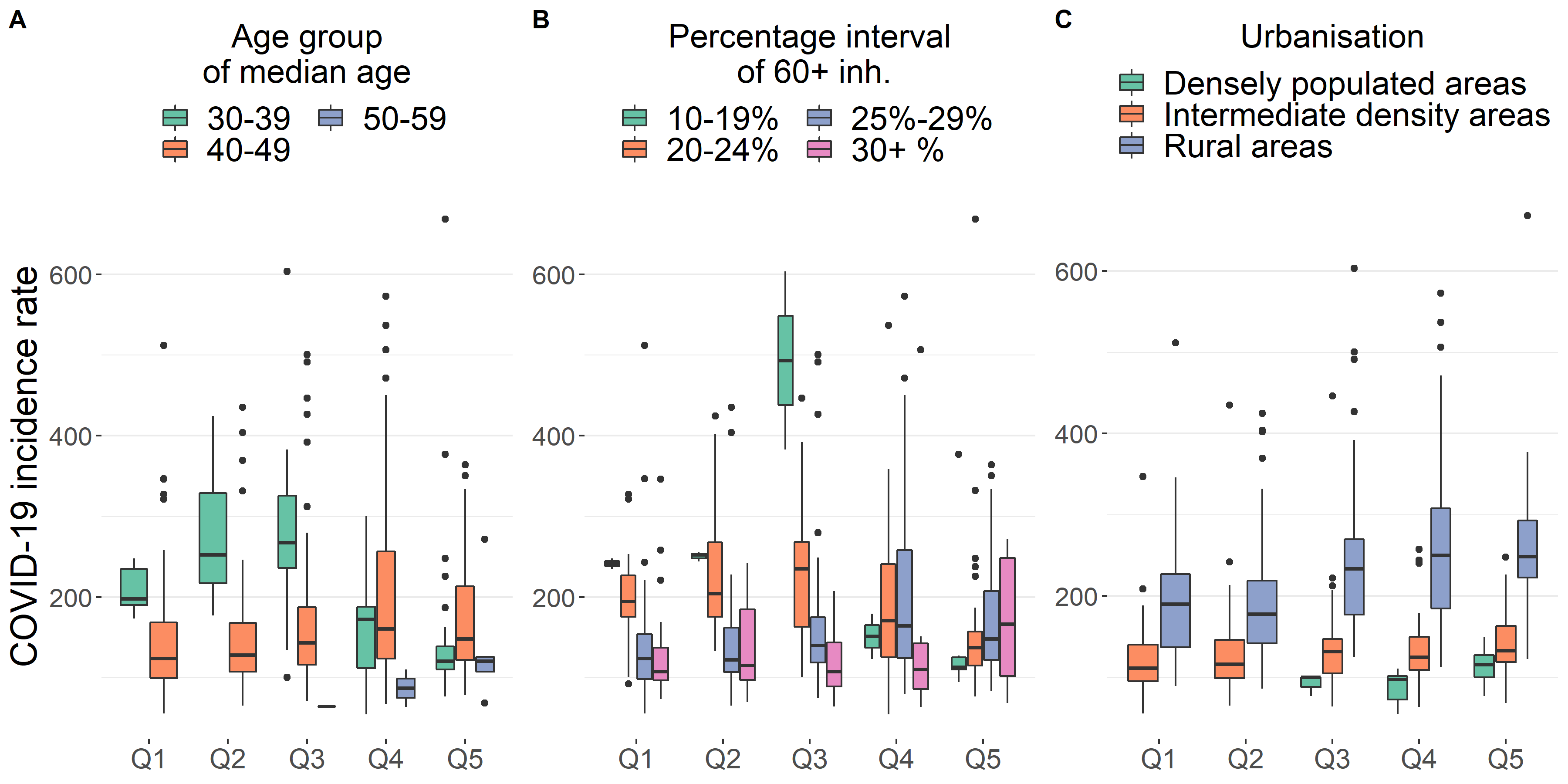

*Supplementary figure 1. Boxplots of the COVID-19 incidence rate (1 March 2020 – 1 June 2021, Belgium), or median weekly number of COVID-19 cases per 100,000 inhabitants, with the area deprivation by (A) median age interval for each quintile (Q1-Q5), (B) the percentage of people older than 60 years old within a given interval for each quintile (Q1-Q5) and (C) the degree of urbanization for each quintile (Q1-Q5) . Q1 represents the least deprived quintile, Q5 the most deprived quintile. *An outlier was removed of a municipality of Q1, with a COVID-19 incidence rate value of 8860,8.*

***
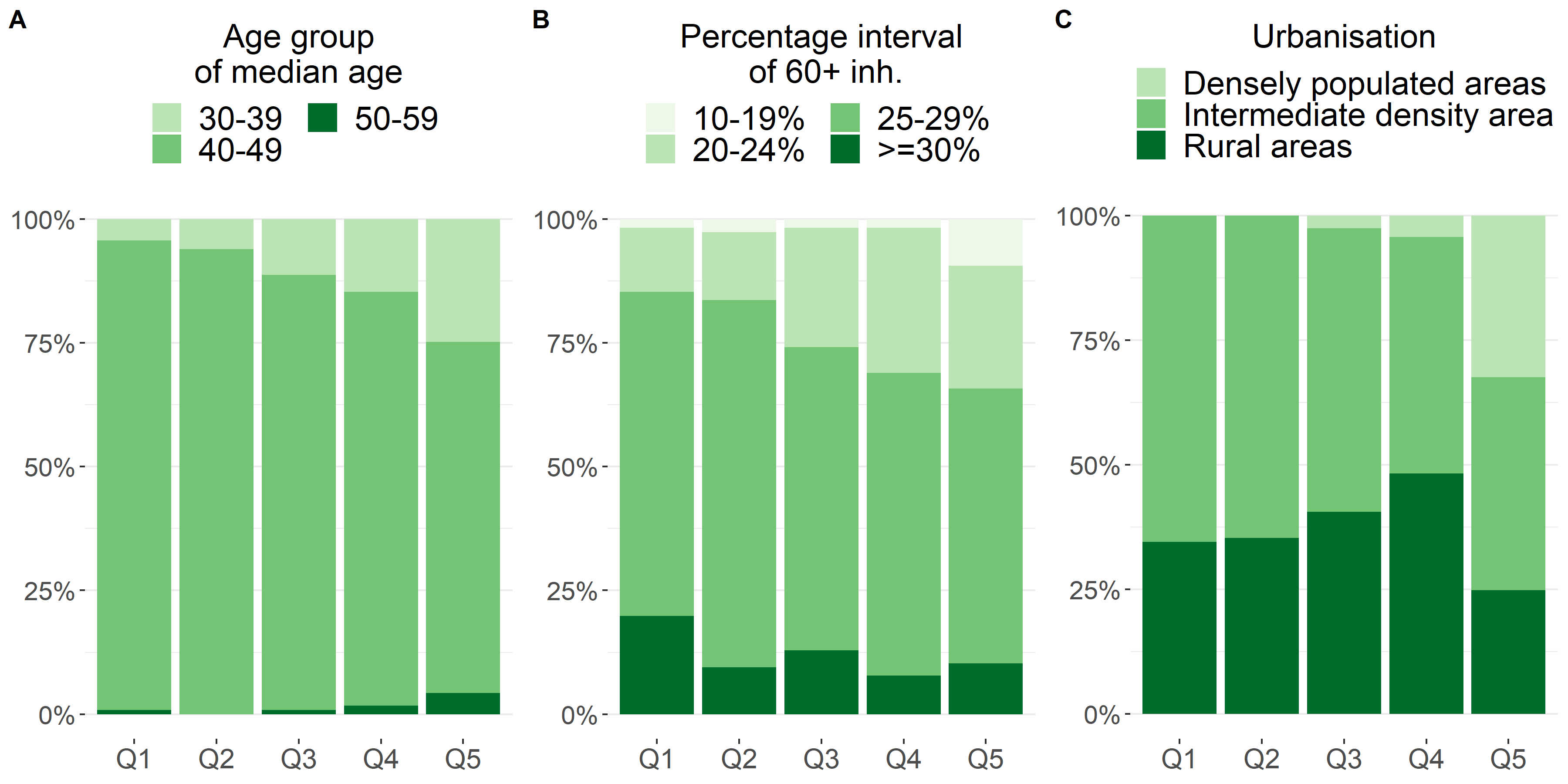
****Supplementary figure 2. The proportion of Belgian municipalities (A) within a given median age interval for each quintile (Q1-Q5), (B) for which the percentage of people older than 60 years old is within a given interval for each quintile (Q1-Q5) and (C) with a given degree of urbanization for each quintile (Q1-Q5). Q1 represents the least deprived quintile, Q5 the most deprived quintile.*


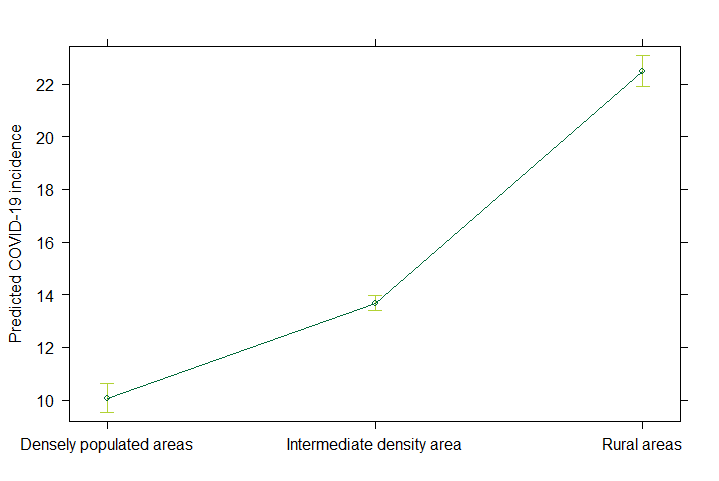
*Supplementary figure 3. Predicted COVID-19 incidence (1 March 2020 - 1 June 2021, Belgium) from the negative binomial regression in function of the degree of urbanization (i.e., Densely populated areas, Intermediate density areas and Rural areas). Municipality-specific population size was used as an offset variable and we controlled for the median age per municipality and area deprivation in the regression model.*
